# A Comprehensive Overview of Online Resources to Identify and Predict Bacterial Essential Genes

**DOI:** 10.3389/fmicb.2017.02331

**Published:** 2017-11-27

**Authors:** Chong Peng, Yan Lin, Hao Luo, Feng Gao

**Affiliations:** ^1^Department of Physics, School of Science, Tianjin University, Tianjin, China; ^2^Key Laboratory of Systems Bioengineering (Ministry of Education), Tianjin University, Tianjin, China; ^3^SynBio Research Platform, Collaborative Innovation Center of Chemical Science and Engineering (Tianjin), Tianjin University, Tianjin, China

**Keywords:** essential gene, minimal gene set, gene essentiality prediction, synthetic biology, Tn-seq analysis

## Abstract

Genes critical for the survival or reproduction of an organism in certain circumstances are classified as essential genes. Essential genes play a significant role in deciphering the survival mechanism of life. They may be greatly applied to pharmaceutics and synthetic biology. The continuous progress of experimental method for essential gene identification has accelerated the accumulation of gene essentiality data which facilitates the study of essential genes *in silico*. In this article, we present some available online resources related to gene essentiality, including bioinformatic software tools for transposon sequencing (Tn-seq) analysis, essential gene databases and online services to predict bacterial essential genes. We review several computational approaches that have been used to predict essential genes, and summarize the features used for gene essentiality prediction. In addition, we evaluate the available online bacterial essential gene prediction servers based on the experimentally validated essential gene sets of 30 bacteria from DEG. This article is intended to be a quick reference guide for the microbiologists interested in the essential genes.

## Introduction

Essential genes are those that play a decisive role in the survival and development of an organism under general conditions. Even though the genome sizes and gene compositions differ dramatically, all so far sequenced genomes contain a set of essential genes that sustain key cellular functions. However, the phrase “essential gene” is highly context-dependent. Only when the environment in which organisms live is clearly defined can a gene be classified as essential gene or not. Another closely linked concept is the minimal gene set. A minimal gene set is defined as the minimal set of genes needed for a cell to carry out basic metabolism and reproduction under the most favorable conditions, in which all essential nutrients are available and there is no environmental stress (Koonin, 2000, 2003; Gil et al., 2004). Research on essential genes, with important theoretical as well as practical values, is quite appealing. Identification of essential genes can help a lot in deciphering the survival mechanisms of life. Moreover, because the deletion or inactivation of essential genes confer lethal phenotypes to microorganisms, essential genes or proteins encoded by essential genes form logical targets for new antibiotics in the pharmaceutical industry (Galperin and Koonin, 1999; Juhas et al., 2011; Mobegi et al., 2014). In the emerging scientific field of synthetic biology, devising a minimal genome is a desirable research direction (Pei et al., 2011; Juhas et al., 2012). For example, researchers at the J. Craig Venter Institute (JCVI) produced the first self-replicating synthetic cell *Mycoplasma mycoides* JCVI-syn1.0 in 2010 (Gibson et al., 2010). By the design-build-test (DBT) cycle, they removed non-essential genes in JCVI-syn1.0 genome and produced JCVI-syn3.0. Containing 531,560 base pairs and only 473 genes, JCVI-syn3.0 has smaller genome than that of any free-living organism found in nature (Hutchison et al., 2016).

Since 1999, when the first global transposon mutagenesis was performed on *Mycoplasma genitalium* to experimentally confirm the minimal gene set for a living organism (Hutchison et al., 1999), the attempt to search for essential genes has been persistently carried out in a wide range of species. The experimental approaches used to identify essential genes include single-gene knockout (Kobayashi et al., 2003), transposon mutagenesis (Hutchison et al., 1999), and antisense RNA inhibition (Ji et al., 2001). In the past decade, the integration of transposon mutagenesis and high-throughput sequencing has facilitated many methods in the recognition of essential genes. These development lead to a significant increase in the number of species involved in gene essentiality screens. Apart from bacteria, essential genes in archaea (Sarmiento et al., 2013) and eukaryotes such as *Saccharomyces cerevisiae* (Giaever et al., 2002), *Schizosaccharomyces pombe* (Kim et al., 2010), *Arabidopsis thaliana* (Meinke et al., 2008), *Mus musculus* (Liao and Zhang, 2007) and *Homo sapiens* (Blomen et al., 2015; Wang et al., 2015) are all identified. Based on these abundant data, researchers have constructed many essential gene databases. The bioinformatic resources greatly promote the investigation of essential genes (Lin et al., 2010; Gao and Zhang, 2011; Peng and Gao, 2014; Luo et al., 2015; Zhang et al., 2015; Zheng et al., 2015).

**FIGURE 1:**
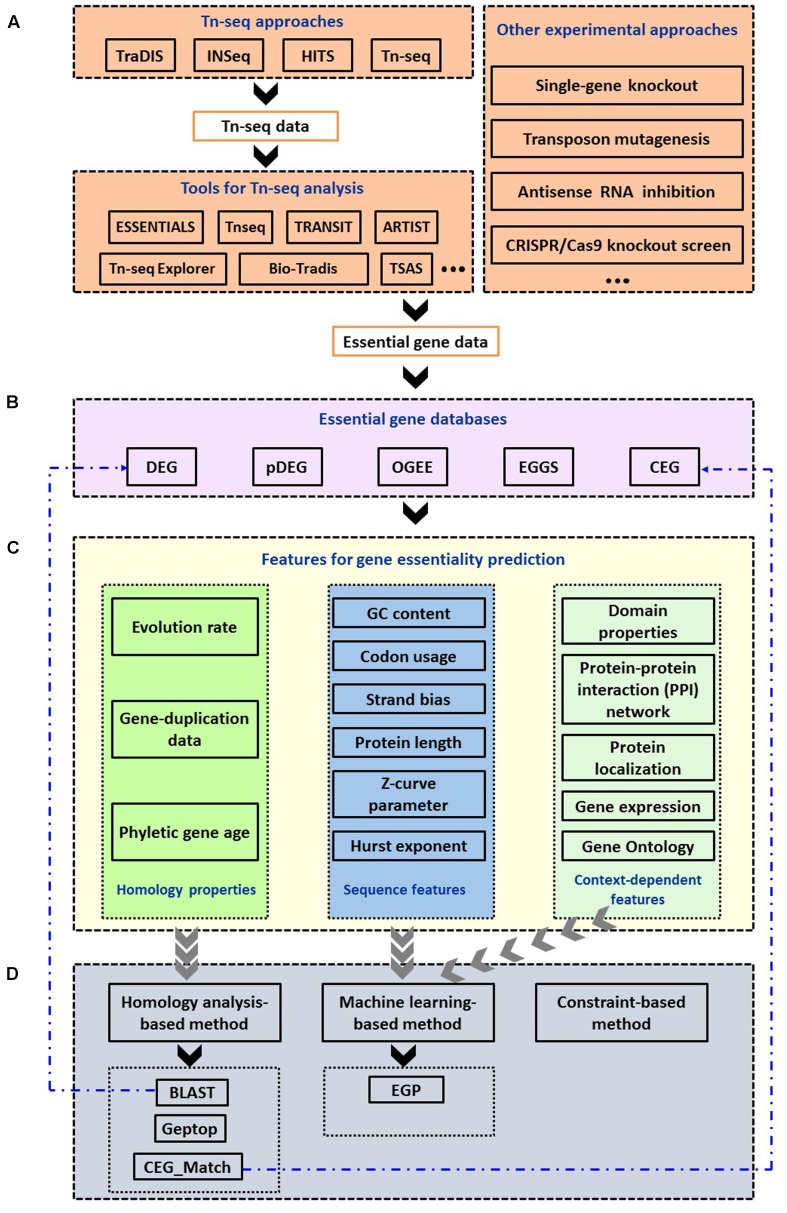
Summary of computational methods used in the identification and prediction of bacterial essential genes. **(A)** Experimental approaches and bioinformatic tools to identify essential genes. **(B)** Essential gene databases. **(C)** Features used for gene essentiality prediction. **(D)** Computational approaches to predict essential genes and online services.

Except the development of experimental approaches, researchers also tried in many ways to computationally recognize the essential genes. In fact, computational approach to search for the minimal gene set was performed as early as 1996. Supposing that genes conserved between organisms are likely to be essential, Mushegian and Koonin compared genomes of *Haemophilus influenzae* and *Mycoplasma genitalium* to determine the minimal gene set (Mushegian and Koonin, 1996). In the past few years, the accumulation of completely sequenced bacterial genomes and the establishment of essential gene database greatly facilitated the identification of bacterial gene essentiality *in silico*. Computational methods are becoming more important in essential gene study because they can dramatically save time and efforts. This article is a comprehensive overview of online resources to identify and predict bacterial essential genes. We present some available web resources related to gene essentiality, including the bioinformatic tools and databases. We also summarize several features used in essential gene prediction. In the final part, the currently available online bacterial essential gene prediction servers are listed and tried based on the experimentally validated essential gene sets of 30 bacteria for evaluation. **Figure 1** shows the outline of this article.

## Experimental Approaches and Bioinformatic Tools to Identify Essential Genes

Previous experimental approaches used to identify essential genes include the systematic inactivation of each individual gene present in a genome, the use of antisense RNA to inhibit gene expression and massive transposon mutagenesis (the most widely used approach) (Gil et al., 2004). Briefly, the single-gene knockout strategy is designed to insert a non-replicating plasmid into the target gene via a single crossover recombination, which is able to disrupt the function of the target gene and generate knockout mutations. The gene that could not be inactivated by insertion is deemed essential (Kobayashi et al., 2003). Antisense RNA inhibition method decreases the expression level of a target gene through binding by double-stranded RNA (dsRNA) (Ji et al., 2001). Another method, transposon mutagenesis is used to identify essential genes by constructing a random transposon-insertion library, then determining the insertion sites by DNA hybridization (Hensel et al., 1995) or microarray (Mazurkiewicz et al., 2006). However, these experimental methods have limitations more or less. The single-gene knockout strategy requires detailed genome annotation. The use of antisense RNA is limited to the genes for which an adequate expression of the inhibitory RNA can be obtained in the organism under study. Shortcomings of transposon mutagenesis method include missing low-abundance transcripts, low resolution in locating insertion sites, and narrow ranges in counting probe density. Therefore, these methods have only been performed in a limited number of organisms and identified their essential genes with low throughput (Gil et al., 2004).

In recent years, technologies that use a random transposon mutant library followed by next-generation sequencing such as transposon-directed insertion site sequencing (TraDIS) (Langridge et al., 2009), insertion sequencing (INSeq) (Goodman et al., 2009), high-throughput insertion tracking by deep sequencing (HITS) (Gawronski et al., 2009) and transposon insertion site sequencing (Tn-seq) (van Opijnen et al., 2009; van Opijnen and Camilli, 2013) are becoming powerful tools to facilitate high-throughput identification of essential genes. Currently, several bioinformatic software tools have been built and maintained by different research groups, which help researchers to analyze the data from transposon insertion sequencing experiments. A list of Tn-seq data analysis software tools related to essential genes is presented in **Table 1**. Most of these tools are included in the manually curated meta-database OMICtools (Henry et al., 2014).

**Table 1:** Software tools to analyze transposon insertion sequencing data for identifying essential genes.

Tool	Description	Programming language	Availability	Applicated organisms	Reference
ESSENTIALS	An open source, web-based software tool for rapid analysis of high throughput transposon insertion sequencing data	Perl and R	Web-interface: http://bamics2.cmbi.ru.nl/websoftware/essentials/	*Neisseria meningitidis* (Capel et al., 2016)	Zomer et al., 2012
			Source code: http://trac.nbic.nl/essentials/	*Pseudomonas aeruginosa* PAO1 (Turner et al., 2015)	
				*Streptococcus pneumoniae* R6, *Streptococcus pneumoniae* TIGR4, *Haemophilus influenzae* 86 028NP, *Haemophilus influenzae* Rd KW20 and *Moraxella catarrhalis* BBH18 (Mobegi et al., 2014)	
				*Acinetobacter baumannii* ATCC 17978 (Wang et al., 2014)	
				*Streptococcus pneumoniae* (Verhagen et al., 2014) *Streptococcus agalactiae* (Hooven et al., 2016)	
Tnseq	A zero-inflated Poisson model for insertion tolerance analysis of genes based on Tn-seq data	R	http://github.com/ffliu/TnSeq	–	Liu et al., 2016
Tn-HMM	A method for analyzing Tn-Seq data using Hidden Markov Models	Python	http://saclab.tamu.edu/essentiality/HMM/	*Yersinia pestis* (Palace et al., 2014)	DeJesus and Ioerger, 2013
Bayesian analysis method	A Bayesian model to analyze gene essentiality based on sequencing of transposon insertion libraries	Python	http://saclab.tamu.edu/essentiality/	*Streptococcus pyogenes* (Le Breton et al., 2015)	DeJesus et al., 2013
TRANSIT	A software tool for Himar1 Tn-Seq analysis	Python	https://github.com/mad-lab/ transit	–	DeJesus et al., 2015
ARTIST	Analysis of high-resolution transposon-insertion sequences technique	Matlab	http://journals.plos.org/plosgenetics/article?id=10.1371/journal.pgen.1004782#s4	*Shigella flexneri* 2a 2457T (Freed et al., 2016)	Pritchard et al., 2014
				*Staphylococcus aureus* (Santiago et al., 2015)	
Tn-seq Explorer	A package of tools for exploration of the Tn-seq data	Java	http://www.cmbl.uga.edu/downloads/programs/Tn_seq_Explorer/ or	*Streptococcus agalactiae* (Hooven et al., 2016)	Solaimanpour et al., 2015
			https://github.com/sina-cb/Tn-seqExplorer			
Bio-Tradis	A set of tools to analyze the output from TraDIS analyses	Perl and R	https://github.com/sanger-pathogens/Bio-Tradis	–	Barquist et al., 2016
TSAS	Tn-seq analysis software	Java	https://github.com/srimam/TSAS	*Rhodobacter sphaeroides* (Burger et al., 2017)	Burger et al., 2017
TnseqDiff	Identification of conditionally essential genes in transposon sequencing studies	R	https://CRAN.R-project.org/package=Tnseq	–	Zhao et al., 2017


**Table 1** shows that several software tools, especially ESSENTIALS (Zomer et al., 2012), have been successfully applied to the genome-wide essential genes screens in many microorganisms. ESSENTIALS uses the Negative Binomial distribution statistical model to quantify the statistical significance of essential regions. It adopts many data preprocessing steps such as data filtering and normalization as well as post-processing steps to optimize the gene essentiality prediction. ESSENTIALS provides both source code and web-interface, so that researchers with no previous computational experience can analyze the Tn-seq data (Chao et al., 2016). Tn-seq Explorer utilizes a sliding window approach which counts insertions in overlapping windows of a specific size. Regions that are significantly underrepresented in read counts compared with the rest of the genome are identified as essential genes or possibly other essential genomic segments (Solaimanpour et al., 2015). This approach can identify essential genes with a high-resolution. However, when the window size decreases and the number of windows increases, the operational quantity will be magnified (Chao et al., 2016). Algorithms in other software include models using the Poisson distribution (Liu et al., 2016), Bayesian analysis method (DeJesus et al., 2013) and Hidden Markov Models (DeJesus and Ioerger, 2013; Pritchard et al., 2014). TRANSIT, a pipeline for analyzing Himar1 Tn-seq data was developed in 2015. This tool provides two different statistical methods (Bayesian/Gumbel Method and Hidden Markov Model) to identify essential genes in individual datasets and a resampling method to identify conditionally essential genes between different growth conditions (DeJesus et al., 2015). The various statistical methods and the graphical interface make TRANSIT an effective and convenient Tn-seq data analysis tool. However, TRANSIT only offers automatic observation on libraries generated by using the Himar1 transposon. When analyzing other TnSeq libraries, a pre-processor is needed to modify the format of data files. TnseqDiff is a parametric method which uses insertion-level data to identify conditionally essential genes. This method is able to deal with data with multiple experimental conditions (Zhao et al., 2017). Bio-Tradis is a novel software tool for analyzing the output of TraDIS analyses. The provided service is similar to that in Tn-seq Explorer and TRANSIT. Better yet, this is a command-line driven approach which allows the simultaneous processing of many sequencing libraries (Barquist et al., 2016).

More recently, the CRISPR-Cas9 technology has also been used to identify essential genes (Wang et al., 2015; Morgens et al., 2016). Clustered regularly interspaced short palindromic repeats (CRISPRs), together with CRISPR-associated (Cas) proteins, provide bacteria with adaptive immunity to viruses and plasmids (Barrangou and Doudna, 2016). In the CRISPR-Cas9 system, single guide RNAs (sgRNAs), which retain a sequence complementary to the targeted region, direct Cas9 endonucleases to induce a site-specific double-strand break in the DNA. Then the double-strand break is repaired by non-homologous end-joining (NHEJ). Thus, the CRISPR system is able to knockout genes at DNA level (Doudna and Charpentier, 2014). Compared with other methods, CRISPR-based methods have features of low noise, minimal off-target effects and consistent activity across reagents (Evers et al., 2016). Currently, this method is mainly adapted to mammalian cell lines. Therefore, we have not discussed its details in this article.

## Bioinformatic Databases About Essential Genes

By utilizing the experimental approaches and bioinformatic tools, researchers are able to quickly and accurately identify essential genes in a wide range of microorganisms under different experimental conditions. Experimentally screened essential gene data are constantly accumulating. These dramatically increasing data form the foundation of the development of secondary databases about essential genes. In the following part, we list some available web resources and servers related to essential genes and discuss them in detail.

DEG (a database of essential genes) is a comprehensive platform for essential genes. This database was constructed in 2004 and has been updated constantly. The newly released DEG 10 contains a considerable number of essential and non-essential genes in archaeal, bacterial and eukaryotic organisms determined under different environments. Non-essential genes can also be determined in many genome-wide essentiality screens. For the genes whose essentialities are undefined due to the limitation of the experiments, they can neither be classified as essential genes nor as non-essential genes. So non-essential genes are not always the complementary set of essential genes and vice versa. Other essential genomic elements such as essential non-coding RNAs, regulatory sequences, essential promoters and even replication origins are also included. In addition, users are allowed to perform homology searches with the embedded BLAST tool provided in the database. Single genes, multiple genes, annotated genomes and even unannotated genomes can be submitted to DEG for BLAST searches (Zhang et al., 2004; Zhang and Lin, 2009; Luo et al., 2014). The timely updated information and practical tool in DEG make it the most widely used database about essential genes.

Lin and Zhang (2011) developed an essential gene prediction algorithm by integrating the information of biased distribution of essential genes in leading and lagging strands, homologous search and codon adaptation index (CAI) values. The algorithm takes 310 and 379 essential genes in *Mycoplasma pulmonis* UAB CTIP and *Mycoplasma genitalium* G37 contained in DEG as training set. The prediction accuracy in self-consistence and cross-validation tests are 80.8 and 78.9% respectively. 5880 essential genes were predicted by this prediction algorithm in 16 *Mycoplasma* genomes. The predicted genes were then stored in a database of predicted Essential Genes (pDEG). Many detailed information of the predicted essential genes are provided in the database, and the records can be freely accessed and downloaded (Lin and Zhang, 2011).

OGEE is an Online GEne Essentiality database. Both essential and non-essential genes obtained from large-scale experiments are openly accessible in this database. The developers also complement their data with text-mining results. For each gene, a list of associated gene properties, such as gene duplication status, evolutionary origins of the gene, expression profiles and conservation across species, is also collected. It has been proved in a series of studies that these gene properties can affect gene essentiality. The database offers an integrated online tool. Genes can be divided into different groups according to gene properties including whether a gene is a duplicate or singleton and whether a gene is involved in development. Then the proportion of essential genes in each group can be visualized by this tool. In 2016, a new version of OGEE was developed, and new species as well as new datasets were added. Moreover, as DEG the developers reorganized 16 essential gene datasets from 9 human cancers. Users can know whether a gene is shared within different cancer types or is essential in one particular cancer type with OGEE. OGEE is a useful tool for researchers to study the essentiality of genes (Chen et al., 2012a, 2017).

EGGS (Essential Genes on Genome Scale) is a database that holds microbial gene essentiality data which are acquired from genome-wide essential gene selections. Microbial genes are classified into three categories: essential (E) genes, non-essential (N) genes and ‘undefined’ (U) for all other genes. Essentiality data of each gene can be browsed in a gene/protein page. In the EGGS database, users can also visualize and analyze essentiality data in the context of a Subsystem spreadsheet or on a Subsystem diagram. The collection of annotated Subsystems makes the comparative analysis of these data possible, which greatly facilitates the interpretation and application of essentiality data (Overbeek et al., 2005; Gerdes et al., 2006).

CEG is a database of essential gene clusters. This database is available at http://cefg.cn/ceg/. The developers obtained the data of essential genes from DEG. The difference is that essential genes with the same functions are stored in one orthologous cluster. The size of an essential gene cluster can show whether the gene is shared among many species or is species-specific. These cluster properties are of great help in evolutionary research and drug target discovery. The CEG database also provides a prediction tool CEG_Match to predict essential genes based on standard gene names, which is discussed in detail later (Ye et al., 2013).

**Table 2** shows the basic information about the above four databases that store essential genes in the form of single genes. DEG and OGEE contain more species and are updated periodically. It is advised to use these resources as primary ones.

**Table 2:** The basic information of essential gene databases.

Database	Data sources	Species	Category	Bacteria	Archaea	Eukaryotes	Non-coding	Additional tool	URL
DEG	Experiment	43	Essential	15,750(33)^a^	519(1)	33,989(9)	680(6)	BLAST tools to perform species- and experiment-specific BLAST searches for a single gene, a list of genes, annotated or unannotated genomes.	http://tubic.tju.edu.cn/deg/ or http://www.essentialgene.org/
			Non-essential	109,187(32)	1,077(1)	3,573(1)	–		
pDEG	Prediction	16	Essential	5,880(16)	–	–	–	–	http://tubic.tju.edu.cn/pdeg/
OGEE	Experiment and text-mining	48	Essential	21,914(39)	–	16,066(9)	–	Tools in the ‘Analyze’ page to visualize the PE% (proportion of essential genes) as a function of other gene properties, including whether a gene is a duplicate or singleton and whether a gene is involved in development.	http://ogee.medgenius.info
			Non-essential	78,075(29)	–	51,744(8)	–
EGGS	Experiment	11	Essential	5,655(11)	–	–	–	Subsystem spreadsheet and Subsystem diagram.	http://www.nmpdr.org/FIG/eggs.cgi
			Non-essential	27,201(8)	–	–	–	


*^a^The number outside bracket is the amount of essential or nonessential genes in the corresponding item, whereas in bracket is the species of the organisms in the corresponding item.*

## Computational Methods for the Prediction of Essential Genes

### Homology Search and Evolutionary Analysis-Based Methods

Primal efforts to computationally identify essential genes adopted comparative genomic analysis based on sequence homology. Researchers tried to predict the minimal gene set by comparing the first sequenced genomes of *Haemophilus influenzae* and *Mycoplasma genitalium*, and identified 256 candidate essential genes (Mushegian and Koonin, 1996). The ideology for homology mapping methods is simple, i.e., genes shared by distantly related organisms are likely to be essential (Koonin, 2003). With the completion of more bacterial genomes’ sequencing, researchers tried to analyze bacterial genome data in different strains of a single species. Comparative genomic analysis including core genes identification (Zafar et al., 2002) has been successfully implemented to infer the essential genes from the pan-genome of bacterial species such as *Mycoplasma* (Liu et al., 2012), *Liberibacter* (Fagen et al., 2014), *Plasmodium falciparum* (Rout et al., 2015) and *Brucella* spp. (Yang et al., 2016). The evolutionary rate of essential genes is slower than that of non-essential genes. So essential genes are more evolutionarily conserved in bacteria (Jordan et al., 2002; Luo et al., 2015). Other homology properties such as gene-duplication data and phyletic gene age have also been used in the prediction of essential genes. Duplicated genes are also called paralogs. Function and expression of these paralogs often overlap with each other. Duplicated genes are less likely to be essential than singletons because deleting one of the duplicates is not lethal to an organism (Jordan et al., 2002; Chen and Xu, 2005). Genes with more recent phyletic origins (younger genes) are less likely to be essential than that with earlier phyletic origin (older genes). For genes of the same age, singletons are more likely to be essential than duplicates (Chen et al., 2012b). Homology mapping can be used to predict essential genes based solely on genomic sequences. However, this method is limited to conserved orthologs between different species, which often make up only a small percentage of the genomes (Bruccoleri et al., 1998). Moreover, although essential genes tend to be highly conserved, the conserved genes across species are not always essential.

### Machine Learning-Based Methods

Machine learning-based method is another widely used approach to predict essential genes. This method identifies essential genes by constructing and training a classifier according to the features of known essential and non-essential genes. Then the classifiers are applied to the same or other genomes (Zhang et al., 2016). For example, Chen and Xu found the significant correlation between the gene essentiality and its evolutionary rate, gene-duplication rate, its connectivity in protein-protein interaction network and gene-expression cooperativity. By methods of neural network and support vector machine, they predicted gene essentiality of high-throughput data in yeast *Saccharomyces cerevisiae* (Chen and Xu, 2005). Machine-learning algorithms used to train the classifier include support vector machine (SVM), neural network, decision tree, Naïve Bayes model, feature-based weighted Naïve Bayes model (FWM) (Cheng et al., 2013; Ning et al., 2014), and so on. With the advancement in research, a variety of genomic and protein features have been analyzed and used in gene essentiality prediction studies. Generally, the features can be broadly classified into two groups: sequence derived features and context-dependent features (Wang et al., 2013; Mobegi et al., 2016).

#### Sequence Derived Features of Essential Genes

(1) GC content. DNA with high GC content is believed to be more robust and stable (Seringhaus et al., 2006).
(2) Codon usage. The codon usage of essential genes suffers from more evolutionary constraints than non-essential genes (Jordan et al., 2002).
(3) Strand bias. Essential genes tend to be encoded on the leading strand of the chromosome (Lin et al., 2010; Rocha and Danchin, 2003).
(4) Protein length. Although protein length tends to become longer through evolution, essential genes, compared to non-essential genes, have a significantly higher proportion of large and small proteins relative to medium-sized proteins (Lipman et al., 2002; Gong et al., 2008).
(5) Z-curve parameter. The Z-curve theory is a bioinformatic algorithm to display base composition distributions along DNA sequences (Zhang and Zhang, 1994; Zhang, 1997; Gao and Zhang, 2004). All the information that a given DNA sequence carries is included in the corresponding Z-curve. So Z-curve features can be used as sequence derived features for essential gene prediction (Song et al., 2014; Lin et al., 2017). Based on the Z-curve theory, Guo et al. (2017) created a λ-interval Z-curve, which considered the interval range association. They then built a support vector machine-based model to predict human gene essentiality with the λ-interval Z-curve, and obtained excellent performance (Guo et al., 2017).
(6) Hurst exponent. The Hurst exponent is a characteristic parameter which describes the degree of self-similarity of a data set. For genes of similar length, the average Hurst exponent of essential genes is smaller than that of non-essential genes (Zhou and Yu, 2014).

#### Context-Dependent Features of Essential Proteins

(1) Domain properties. Protein essentiality is not likely to be conserved through the conservation of overall proteins but through the function of protein domains or domain combinations (Deng et al., 2011).
(2) Protein-protein interaction (PPI) network. Genes or their protein products are connected rather than isolated. Compared with non-essential genes, essential genes tend to be more highly connected in protein interaction networks. Network topology features, such as degree centrality (DC), betweenness centrality (BC), closeness centrality (CC), eigenvector centrality (EC), subgraph centrality (SC) have been used for detecting essential proteins (Estrada, 2006; Acencio and Lemke, 2009; Hwang et al., 2009; Wang et al., 2013; Xiao et al., 2015).
(3) Protein localization. Essential proteins exist in cytoplasm with a higher proportion, while locate in cell envelope such as cytoplasm membrane, periplasm, cell wall and extracellular with a much lower proportion compared with non-essential proteins (Seringhaus et al., 2006; Peng and Gao, 2014).
(4) Gene expression. Genes whose expression levels are higher and stabler under given conditions are more likely to be essential (Jansen et al., 2002).
(5) Gene Ontology. The Gene Ontology (GO) project provides a set of hierarchical controlled vocabularies for describing the biological process, molecular function, and cellular component of gene products (Ashburner et al., 2000). GO terms related to cellular localization and biological process are shown to be reliable predictors of essential genes (Acencio and Lemke, 2009).

Compared with homology mapping, the supervised machine learning-based methods use more genomic and protein features to construct the predicting model. The prediction performance can be improved by selecting appropriate features (Deng et al., 2011; Lu et al., 2014). However, multiple available gene features lead to complexity as well. Different combinations of features may influence the prediction performance. The prediction results in different organisms with the same feature combinations could also be different. How to select suitable features for the organism under study to accurately predict essential genes is still a question (Mobegi et al., 2016). Another limitation of machine learning-based methods is that they may not be suitable for conditionally essential genes prediction.

### Constraint-Based Approaches

Genome-scale metabolic networks, which help to understand the systems biology of metabolic pathways within an organism, have been reconstructed based on the genomic sequencing and annotations (Thiele and Palsson, 2010). The structure and function of these networks can be studied by constraint-based modeling methods. Constraint-based modeling uses a series of constraints to describe a biological system and characterize its possible behavior under specific environmental conditions (Edwards et al., 2002; Price et al., 2003; Orth et al., 2010). Constraint-based models have been reconstructed in organisms across all three domains of life. These models have promoted the investigation of gene essentiality (Joyce and Palsson, 2008).

Flux balance analysis (FBA) is the most widely used constraint-based approach to analyze the properties of metabolic networks. This approach allows the prediction the metabolite fluxes at steady state by applying mass balance constraints to a stoichiometric model (Kauffman et al., 2003; Raman and Chandra, 2009; Orth et al., 2010). The basic idea of applying FBA to predict essential genes is to simulate the knockout of a gene, and then evaluate the associated lethality on the system (Basler, 2015). Usually, the building and analysis procedure of FBA model can be divided into three steps. First, reconstruct the metabolic network and compile the stoichiometric matrix. Second, identify and apply appropriate constraints to the network. Finally, find the optimal flux distribution by linear programming and assess the essentiality of a gene through analysis of the optimal flux distribution in the network (Joyce and Palsson, 2008; Lu et al., 2014; Basler, 2015). FBA is less computationally expensive because it does not require kinetic parameters. FBA can be used to perform the simulation of large numbers of perturbations to the network. This approach is suitable for conditionally essential gene studies. However, it cannot be used to predict metabolite concentrations or transient dynamic states because it does not use kinetic parameters. Furthermore, the predictions sometimes disagree with experimental data, because FBA does not account for regulatory effects such as regulation of gene expression (Orth et al., 2010; Basler, 2015). Nevertheless, FBA has obvious limitations because it could only predict the essentiality of a metabolic gene.

## Evaluation of Online Essential Gene Prediction Servers

The CEG_Match is developed based on the CEG database. It is a gene essentiality prediction tool based on their functions. The CEG_Match predicts essential genes by matching the standard gene names and the cluster names stored in the CEG database. Compared with direct blast search against CEG database, this methodology is more accurate because there are no obvious similarities between two genes with different functions, while two genes without obvious similarities may have the same function. Users should input gene names in a one name per line format or gene sequences in fasta format. They are also required to adjust the minimum matching number before executing the tool. Generally, it’s more likely for the gene to be essential if the matching number is larger. However, the CEG_Match tool has its limitations. It works only when the gene name is known (Guo et al., 2010, 2015; Ye et al., 2013).

Geptop is a gene essentiality prediction tool for sequenced bacterial genomes based on orthology and phylogeny. A gene is more likely to be essential if it is conserved during the long-term evolution, especially in similar species. The reciprocal best hit (RBH) method was used for estimating orthology. The distance of phylogeny between species was computed with the Composition Vector (CV) method. An open source standalone package version is also offered on the website. Any bacterial species with sequenced genome can get essential gene searched by Geptop. Moreover, the website stored essential genes in 968 bacterial genomes predicted by Geptop. Users can browse and download the data for further research (Wei et al., 2013).

ZCURVE (Guo et al., 2003) is a program that predicts genes in bacterial or archaeal genomes. It is developed based on the Z-curve theory. Its latest version ZCURVE 3.0 has an embedded Geptop program, which has an extended function of searching for essential genes in bacterial or archaeal genomes. However, different from the previous Geptop, predicted genes are used here as the input rather than annotated genes. Once the essential genes output option is selected, users can get an output file showing whether each predicted gene is essential or not (Hua et al., 2015).

EGP (Essential Gene Prediction) is an online tool for essential gene prediction of bacteria genomes. It is a support vector machine (SVM)-based method which only uses sequence compositional features. Five groups of features, including amino acid usage, codon usage, nucleotide usage of 3 codon positions, di-nucleotide usage, and CodonW features are independently and jointly input into the SVM to construct the predicting model. The training dataset consists essential genes in 16 bacterial genomes. For large-scale genome sequences, the accuracy of EGP can reach 75%. Users only need to provide nucleotide sequences of genes to make a prediction. The predicted result will be presented on the jumping window or be sent to users by e-mail (Ning et al., 2014).

**FIGURE 2:**
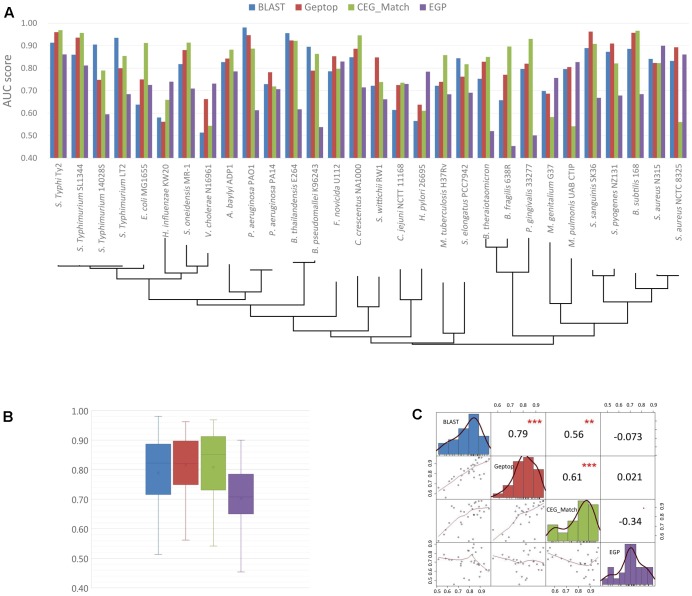
Prediction performance of BLAST tool, cross-organism Geptop, CEG_Match and EGP in the 30 genomes. **(A)** AUC scores of the gene essentiality prediction by BLAST tool, cross-organism Geptop, CEG_Match and EGP incorporating the phylogenetic information of the 30 genomes. **(B)** Box plot of AUC scores from the prediction of the four tools for the 30 genomes. **(C)** Correlation analysis of AUC scores from the prediction of the four tools.

The basic information of the online essential gene prediction servers including CEG_Match, Geptop, ZCURVE 3.0, EGP and BLAST tool in DEG are presented in **Table 3**. The differences in the use of each tool are also listed. Researchers can choose the suitable servers according to actual conditions. We test the prediction performance of BLAST tool, Geptop, CEG_Match and EGP by 30 bacteria, whose experimentally validated essential gene sets are collected in DEG. Protein sequences of both essential and non-essential genes in the 30 genomes are independently uploaded to DEG for homologous searching. At the selecting organism step, all the organisms are selected except the one the query proteins belong to, which enable it to be a cross-organism test. Geptop has the same issue. We abandon the web server and use the standalone version to perform the test. When the tested genome is included in the reference species, the other 18 proteomes are used as the training set. A limitation with CEG_Match is that we can only perform the prediction to the genes with known name. We use the AUC [area under the receiver operating characteristic (ROC) curve] score as the standard method to assess the accuracy of the four predictive tools. The AUC scores are shown in **Figures 2A,B**. The phylogenetic tree was constructed to elucidate the evolutionary relationship among the organisms. The black lines in **Figure 2A** are the phylogenetic tree of the 30 organisms used in the prediction. The tree was constructed by the MEGA6 program (Tamura et al., 2013) with the sequences of 16S ribosomal RNA of the 30 organisms, which are downloaded from the NCBI website. In **Figures 2A,B**, we can see that the prediction accuracy of EGP is lower than the other three tools. **Figure 2C** shows that the prediction accuracy of BLAST tool, Geptop and CEG_Match show positive correlation. For these three tools, if the input species belongs to the same phylogenetic lineage with any of the reference species, the prediction accuracy of this organism is higher. From this we can infer that the accumulation of the experimental data can improve the tools to get better predictions.

**Table 3:** Summary of the online essential gene prediction servers.

Name	Methodology	Input	Standalone version	Annotation	URL
CEG_Match	Based on gene function	Standard gene name	×	The limitation of CEG_Match is that it is only applicable to name known genes. This will be an appropriate tool when you only know the genes’ names and the complete genome is not at hand.	http://cefg.cn/ceg/predict.php
Geptop	Based on orthology and phylogeny	Amino acid sequence	√	Geptop tool could be applicable only when the investigated genomes have been completely sequenced.	http://cefg.uestc.edu.cn/geptop/
ZCURVE 3.0	Based on orthology and phylogeny	Amino acid sequence of predicted genes	√	ZCURVE 3.0 is a program to find genes in bacterial or archaeal genomes. It has an embedded Geptop program, which has an extended function of searching for essential genes.	http://cefg.uestc.edu.cn/zcurve/ or http://tubic.tju.edu.cn/zcurveb/
EGP	Machine learning-based method	Nucleotide sequence	×	The accuracy of EGP is lower than other tools. Before using this tool, it is advised to check the reference species, which have been used in the training set of EGP. Be cautious to use it when your input gene belongs to the host that does not be included in the same family with any of the reference species.	http://cefg.uestc.edu.cn:9999/egp
BLAST	Homology search-based method	Nucleotide sequence Amino acid sequence	√	DEG has a set of customizable BLAST tools to perform homologous searches against essential gene sets in DEG. Single genes, multiple genes, annotated genomes and unannotated genomes can be submitted for BLAST searches.	http://tubic.tju.edu.cn/deg/


## Conclusion and Perspectives

Studies on essential genes are gradually becoming popular and can promote our understanding of biology. They may also be applied to pharmaceutical as well as synthetic biology. Predicting essential genes *in silico* will become more important because computational methods are helpful in reducing the research space for essential gene identification. The computational approaches can be performed only when enough experimental essential genes data are available. The development of many bioinformatic software tools has facilitated the identification of essential genes. The gene essentiality databases have collected such data and contributed a lot in the characterization of essential genes. Multiple computational approaches have been established based on the features proven to be related to gene essentiality, and have made significant advancement in essential gene prediction. In this review, with an emphasis on the online resources, we summarized several computational methods of predicting bacterial essential genes. However, challenges still remain. For example, diverse gene features have been proven to be related to gene essentiality, but finding out true essentiality related features for a given genome is quite complicated. When the prediction methods are applied to a few model organisms, we may usually get favorable results, but when involving more organisms, the results are not so satisfactory. Besides, it is difficult to predict essential genes under different living conditions. For such scenarios, more and better experimental data can trigger the development of enhanced prediction tools.

## Author Contributions

FG conceived and designed the study. CP performed the study and drafted the manuscript. YL and HL took part in the data analysis. All the authors edited the manuscript and approved the final manuscript.

## Conflict of Interest Statement

The authors declare that the research was conducted in the absence of any commercial or financial relationships that could be construed as a potential conflict of interest.
